# Drug-Induced Hypersensitivity Syndrome Caused by Carbamazepine Used for the Treatment of Trigeminal Neuralgia

**DOI:** 10.1155/2016/4605231

**Published:** 2016-11-03

**Authors:** Yuko Ono, Tsuyoshi Shimo, Yoshinori Shirafuji, Toshihisa Hamada, Masanori Masui, Kyoichi Obata, Mayumi Yao, Koji Kishimoto, Akira Sasaki

**Affiliations:** ^1^Department of Oral and Maxillofacial Surgery, Okayama University Graduate School of Medicine, Dentistry, and Pharmaceutical Sciences, Okayama 700-8525, Japan; ^2^Department of Dermatology, Okayama University Graduate School of Medicine, Dentistry, and Pharmaceutical Sciences, Okayama 700-8525, Japan

## Abstract

An 88-year-old man was diagnosed with trigeminal neuralgia, and treatment of carbamazepine 200 mg/day was initiated. About 6 weeks later, the patient developed a skin rash accompanied by fever. He was admitted to hospital and diagnosed with drug-induced hypersensitivity syndrome (DIHS) caused by carbamazepine. Oral carbamazepine treatment was stopped, but blood tests showed acute liver and acute renal failure. Drug-induced lymphocyte stimulation test (DLST) for carbamazepine, human herpes virus-6 (HHV-6) IgG, and CMV-HRP were negative. Oral prednisolone therapy was begun 18 days later. The titer of HHV-6 IgG antibodies was then detected (640 times). Following treatment, liver and renal function improved and the erythema disappeared.

## 1. Introduction

Drug-induced hypersensitivity syndrome (DIHS) is a severe drug eruption characterized by a generalized maculopapular and erythrodermic rash, high fever (≧38°C), lymphadenopathy, hepatic dysfunction, leukocytosis with eosinophilia, and an increased number of atypical lymphocytes, usually appearing 2–6 weeks after exposure to a certain drug [1]. This disease is associated with reactivation of human herpes virus-6 (HHV-6) and/or other herpes viruses such as HHV-7, cytomegalovirus (CMV), and Epstein-Barr virus (EBV) [2]. In this report, we describe a case of carbamazepine-induced erythrodermic rash associated with DIHS, which was suspected with HHV-6 reactivation.

## 2. Case Report

**Figure 1:**
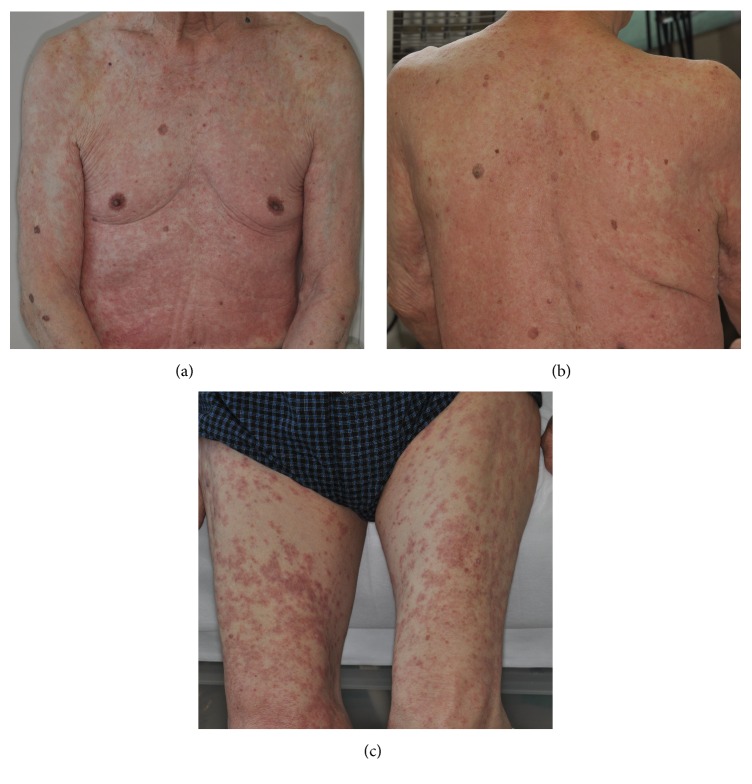
Clinical picture of the patient. Maculopapular rash on the trunk (a, b) and the thighs (c).

In July 2013, an 88-year-old Japanese man was referred to our hospital with a 1-month history of intense, electric-shock-like pain caused by irritation of the right lower jaw. He was diagnosed with right trigeminal neuralgia and started on a treatment of oral carbamazepine 200 mg/d. His symptoms improved. After 6 weeks of medication, he became aware of a body rash with itching. He visited the local clinic where topical corticosteroid and antihistamine were prescribed. When the symptoms had not changed 4 days later, however, he came to our hospital. The carbamazepine was discontinued, and he was sent for a dermatology consult and admitted as a patient. By August 19, he had developed a rash and had multiple areas of exudative erythema on his face, trunk, and limbs (Figures 1(a)–1(c)).

The patient had a fever of 38.2°C, and his blood tests showed liver enzyme elevation [aspartate aminotransferase (AST), 47 U/L; alanine aminotransferase (ALT), 146 U/L; *γ*-glutamyl transferase (*γ*-GTP), 250 U/L] and renal dysfunction [creatinine (CRTN), 2.74 mg/dL].

**Figure 2:**
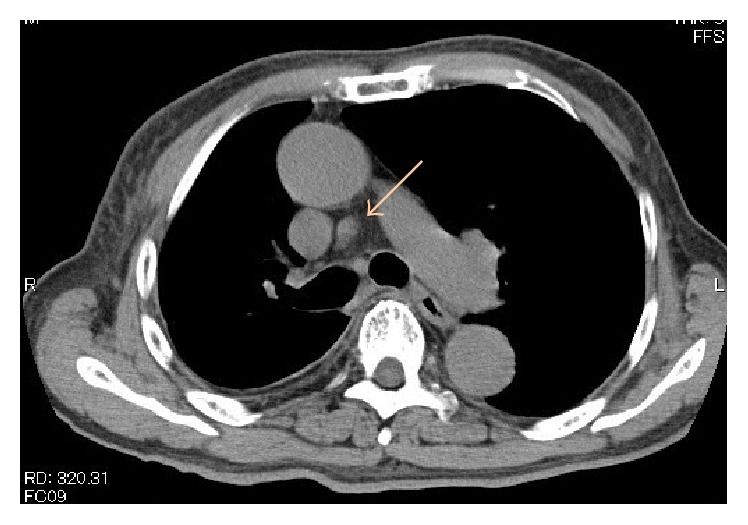
Mediastinum computed tomography.

**Figure 3:**
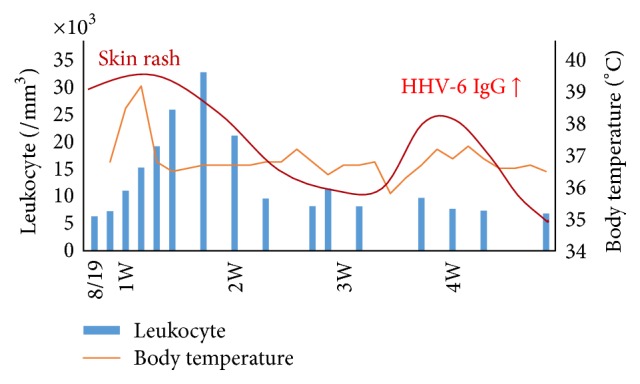
Clinical course of the patient.

Drug-induced lymphocyte stimulation test (DLST) for carbamazepine, HHV-6 IgM, IgG, and DNA and CMV-HRP were negative. Computed tomography (CT) showed lymphadenopathy at the mediastinum (Figure 2). The patient's skin rash worsened and the fever rose to 39.1°C on August 23. Laboratory data showed a white blood cell count (WBC) of 19,210/*μ*L with 22% eosinophils; CRP, 8.34 mg/dL; CRTN, 3.59 mg/dL. He was diagnosed with drug eruption on the basis of clinical symptoms and the results of laboratory data and treated with oral prednisolone 30 mg/d. His blood tests showed a white blood cell count of 32,870/*μ*L with 42% eosinophils, and an increased number of atypical lymphocytes appeared; in response, the prednisolone was increased to 60 mg/d. After treatment, his liver and renal function and rash improved gradually. However, he developed a rash again on his trunk and limbs on September 9. His blood tests showed serum anti-HHV-6 IgG titer was 640 times the normal value (Figure 3). The patient was diagnosed with DIHS according to criteria for DIHS by the presence of the seven categories (see the following list) [3]. After treatment with oral prednisolone and olopatadine hydrochloride 10 mg/d, the erythema disappeared, and the prednisolone was gradually decreased. 

*Diagnostic Criteria for DIHS Established by a Japanese Consensus Group [4]*

(1) Maculopapular rash developed >3 weeks after starting with a limited number of drugs
(2) Prolonged clinical symptoms after discontinuation of the causative drug
(3) Fever (>38°C)
(4) Liver abnormalities (ALT > 100 U/L) (this can be replaced by other organ involvement, such as renal involvement)
(5) Leukocyte abnormalities (at least one present)
  (a) Leukocytosis (>11 *∗*10^9^/L)
  (b) Atypical lymphocytosis (5%)
  (c) Eosinophilia (>1.5 *∗*10^9^/L)
(6) Lymphadenopathy
(7) HHV-6 reactivation

The diagnosis is confirmed by the presence of the seven criteria above (typical DIHS) or of five of the seven (atypical DIHS).

## 3. Discussion

In many cases, the administration period of DIHS is 2–6 weeks. DIHS first came to attention as a new pathology that compounded drug allergies and virus infectious diseases. It has been reported that the prognosis of DIHS may be influenced by age, genetic factors, presence of underlying disease, viral reactivation, and type of treatment. Hepatitis and renal dysfunction are observed during the early phase of DIHS [5]. In this case, liver and renal dysfunction were observed on admission day, August 20. However, it remains unclear what induces these complications. Miyazaki et al. suggested that an immune-mediated reaction in DIHS might generate overactivation of macrophages and T-lymphocytes, followed by a cytokine storm that affects various organs [6]. In future, cytokine measurements such as IL-6 and IL-10 may become one of the factors elucidating the mechanisms of immune reaction that results in pancytopenia.

Carbamazepine is the first-choice standard drug for the treatment of trigeminal neuralgia in the oral maxillofacial field. DIHS is triggered by some restricted drugs, especially carbamazepine. Thus, it is the most frequent cause of DIHS in this country [7]. As to why this is so, the abnormality of the metabolizing enzyme is assumed to be the cause, although the mechanisms are still unknown [8]. After carbamazepine dosage is stopped, a symptom may still progress and require about one month before it is relieved. This case showed two peaks characteristics clinically, and it was revealed from the reports about the course that reactivation of HHV-6 was involved [5]. The rise of the anti-HHV-6 IgG antibody titer was not seen 7 days after symptom onset, but a significant rise in the anti-HHV-6 IgG antibody titer was observed at 25 days after onset. The reactivation of HHV-6 in DIHS happened at the limited time, and these results suggested that they participated in the pathological mechanism closely.

It is important to keep in mind the possibility of drug-induced reactions that might cause acute liver and renal failure [6]. Tohyama et al. reported that patients with serious liver function disorder or serious renal function belonged to the HHV-6 reactivated group; furthermore, the poor prognosis cases belonged to all cases of HHV-6 reactivation group [9]. These results suggested that reactivation of HHV-6 follows the revival of clinical manifestations, protraction, and aggression of DIHS closely. In future, a genetic risk factor for carbamazepine-induced DIHS may provide useful information for decisions regarding individualized medication of anticonvulsants [7]. Oral administration of corticosteroids remains the general cure for the treatment of DIHS [5].

In this case report, we described a patient with DIHS caused by carbamazepine. DIHS is the new disease concept in which drug allergies and viral infections are compounded, and it is important to recognize that viral infection participates in various kinds of diseases including drug-caused skin eruption.
